# Association between *In Vitro* fertilization and gestational diabetes mellitus: a multicenter cohort study

**DOI:** 10.3389/fendo.2026.1754140

**Published:** 2026-03-11

**Authors:** Ping Yu, Qiong Li, Deshen Han, Yu Chen, Ying Gu, Chaoyan Yue

**Affiliations:** 1 Center of Reproductive Medicine, Women’s Hospital of Jiangnan University, Wuxi Maternity and Child Health Care Hospital, Wuxi, China; 2 Department of Obstetrics and Gynecology, The First People’s Hospital of Chenzhou, Chenzhou, China; 3 Wuxi Medical School, Jiangnan University, Wuxi, China; 4 Department of Obstetrics, Wuxi Maternity and Child Health Care Hospital, Wuxi School of Medicine, Jiangnan University, Wuxi, China; 5 Obstetrics & Gynecology Hospital of Fudan University, Shanghai Key Lab of Reproduction and Development, Shanghai Key Lab of Female Reproductive Endocrine Related Disease, Shanghai, China

**Keywords:** assisted reproductive technology, gestational diabetes mellitus, *in vitro* fertilization, metabolic disorders, risk stratification

## Abstract

**Introduction:**

This multicenter retrospective cohort study investigates the association between in vitro fertilization (IVF) and gestational diabetes mellitus (GDM) risk across demographic subgroups.

**Methods:**

Using clinical data from 61,329 pregnant women (3,902 IVF-conceived; 57,427 naturally conceived) across three tertiary hospitals in China, we compared GDM incidence diagnosed through standardized oral glucose tolerance tests (24-28 gestational weeks).

**Results:**

The IVF group exhibited significantly higher GDM incidence (17.32% vs. 12.91%, p<0.001). The analysis adjusted for age, parity, pre-pregnancy body mass index (BMI), family history of DM, alanine aminotransferase (ALT), and creatinine revealed IVF-conceived pregnancies had 15% elevated GDM risk. Subgroup analyses demonstrated heightened IVF-associated GDM risk in women aged <35 years, those with pre-pregnancy BMI <24 kg/m^2^, and primiparous women. No significant associations were observed in older women (≥35 years), individuals with BMI ≥24 kg/m^2^, or multiparous women.

**Discussion:**

These findings identify IVF as an independent GDM risk factor, particularly in younger, leaner, and first-time mothers. The results underscore the importance of stratified prenatal monitoring and mechanistic investigations into fertility treatments’ metabolic impacts.

## 1 Introduction

Infertility has emerged as a significant public health issue worldwide, affecting approximately 10% to 15% of reproductive-age couples (1). Infertility has become one of the most common chronic health issues affecting individuals of reproductive age. The analysis of the epidemiological characteristics of infertility from 1990 to 2021 indicates that the pooled period prevalence of infertility is 12.6%, while the pooled lifetime prevalence is 17.5% (2). Current infertility treatment options encompass a range of approaches, including pharmacological interventions, surgical procedures, and assisted reproductive technologies (ART) (3). Among these, ART often represents a last resort for couples struggling to conceive. In recent years, IVF has gained widespread application and has become one of the primary methods for treating infertility.

Scholars have increasingly focused on the safety of IVF for mothers and infants, with GDM emerging as a significant area of concern. Global prevalence rates for GDM range from 5% to 20% (4). GDM poses a global health challenge, associated with adverse outcomes for both mothers and newborns (5). Existing research indicates that women undergoing ART may have a higher risk of developing GDM compared to those who conceive naturally (5–9). However, a single-center study, after adjusting for confounding factors such as age, parity, and pre-pregnancy BMI, found no significant association between ART and GDM (10). These conflicting findings underscore the ongoing debate regarding the impact of ART as an exposure factor on GDM risk. Such discrepancies may partly arise from the limited data sources and small sample sizes in previous studies, which could introduce bias in the results. Most existing research is constrained by single-center designs or small cohorts and lacks a comprehensive exploration of differences across various age groups, pre-pregnancy BMI, and nulliparous versus multiparous populations.

To address these controversies and provide more reliable conclusions, this study utilizes a large-scale, multicenter clinical dataset to investigate the association between IVF as an exposure factor and the risk of GDM. We employed multivariable logistic regression analysis to assess the independent effect of IVF on the risk of GDM, controlling for various potential confounders. Furthermore, to gain a deeper understanding of the underlying mechanisms, we conducted subgroup analyses to evaluate whether the association between IVF and GDM varies by age, pre-pregnancy BMI, and parity. Through these methodological enhancements, this study aims to improve the reliability and generalizability of the findings while minimizing bias and error, thereby providing new scientific insights into the impact of IVF on GDM risk.

## 2 Methods

### 2.1 Patients and design

This research utilized a retrospective cohort design, drawing data from the Obstetrics and Gynecology Hospital of Fudan University (Shanghai, China; n = 39,328), Wuxi Maternal and Child Health Hospital (Wuxi, China; n = 16,159), and Chenzhou First People’s Hospital (Chenzhou, China; n = 5,842). data were systematically extracted from electronic medical records from 3,902 women who achieved conception via IVF (IVF group) and 57,427 women who conceived naturally (NC group). During this process, all participating women underwent screening for GDM and provided standardized medical histories. Eligibility for inclusion mandated the availability of complete data for all key variables. Exclusion criteria encompassed individuals with chronic hypertension, multiple gestations, pre-existing diabetes (type 1/2), and those lacking complete maternal or neonatal records. Maternal and newborn clinical data were extracted from the Hospital Information System (HIS) and the Laboratory Information Management System (LIS). This research complied with the ethical principles specified in the Declaration of Helsinki (1964, and subsequent amendments) and obtained approval from the ethics committees of the participating institutions. This study was approved by the Ethics Committees of the following institutions: the Obstetrics and Gynecology Hospital of Fudan University (Approval No. 2024-189), Wuxi Maternal and Child Health Hospital (Approval No. 2024-06-1024-54), and Chenzhou First People’s Hospital (Approval No. 2024021). A waiver of informed consent was granted for this research.

### 2.2 Variables and measurements

In this study, the exposure factor was the treatment involving assisted reproductive technology. Laboratory measurements were collected during the initial visit. The analysis adjusted for potential confounding factors, which included age, pre-pregnancy BMI, parity, family history of diabetes, as well as levels of ALT and creatinine.

### 2.3 Outcomes

The primary outcome was GDM, GDM A1defined as GDM controlled by diet and exercise, GDM A2, defined as GDM requiring pharmacotherapy. GDM is defined as glucose intolerance first identified during pregnancy in women who did not exhibit any significant glucose metabolism abnormalities prior to conception. Typically, blood glucose levels return to normal postpartum. GDM is diagnosed via an oral glucose tolerance test (OGTT) conducted between the 24th and 28th weeks of pregnancy. The diagnostic cut points for the 75-g OGTT are as follows: 0 hours, 5.1 mmol/L; 1 hour, 10.0 mmol/L; and 2 hours, 8.5 mmol/L. A diagnosis of GDM is established when any one of these values is met or exceeded. (11, 12).

Secondary outcomes included GDM A2, GDM&PE and large for gestational age (LGA). GDM&PE refers to a condition where a pregnant woman simultaneously meets the diagnostic criteria for GDM and preeclampsia during pregnancy. The diagnosis of preeclampsia requires the presence of newly developed hypertension, defined as a systolic blood pressure of ≥140 mmHg and/or a diastolic blood pressure of ≥90 mmHg, occurring after 20 weeks of gestation, accompanied by new-onset proteinuria or other evidence of end-organ dysfunction. (13). LGA is defined as a neonate’s birth weight exceeding the 90th percentile for its gestational age and gender, based on the growth standards established by the Intergrowth-21st project (14).

### 2.4 Statistical analysis

Continuous variables are expressed as medians (interquartile ranges) for non-normally distributed data and means ± standard deviations (SD) for normally distributed data. Categorical variables are reported as frequencies (%). Adjusted odds ratios (ORs) were calculated using multivariable logistic regression models. The selection of confounding factors for adjustment was based on a combination of clinical relevance and statistical criteria. Potential confounders were considered for inclusion in the multivariable models if they were significantly associated with the outcome (p < 0.10 in univariate analysis) or if their inclusion altered the estimated effect of IVF on GDM (odds ratio) by more than 10%. This approach is consistent with established epidemiological practices for variable selection (15). Stratification was performed based on age, pre-pregnancy BMI, and parity. Additionally, a sensitivity analysis was conducted among subgroups to evaluate the consistency of the association between IVF and GDM. Interaction tests were utilized to examine differences in odds ratios across the analyzed subgroups. Statistical analyses were performed using IBM SPSS (version 21.0; IBM, Armonk, NY) and R (version4.4.2; The R Foundation; https://www.r-project.org). All statistical P-values were two-tailed, with significance set at P < 0.05.

## 3 Results

In this cohort study, a total of 57,427 pregnant women were included in the NC group, while the IVF group comprised 3,902 pregnant women. Among these, 7,411 cases (12.91%) in the NC group were diagnosed with GDM, compared to 676 cases (17.32%) in the IVF group. Of these cases, 852 (3.06%) in the NC group and 71 (4.11%) in the IVF group required pharmacological treatment. The incidence of GDM with preeclampsia (GDM-PE) was 528 (0.92%) in the NC group and 71 (1.82%) in the IVF group. The baseline characteristics of all participants included in the study are presented in **Table 1**.

**Table 1:** Baseline characteristics.

Characteristic	NC n=57427	IVF n=3902	*p*
AGE	31.20 ± 4.15	33.52 ± 4.04	<0.001
BMI	21.80 ± 3.21	22.22 ± 3.20	<0.001
ALT	18.21 ± 16.13	19.23 ± 17.44	0.002
Creatinine	40.65 ± 10.40	40.91 ± 10.77	0.191
PARITY			<0.001
Primigravida	38770 (68.65%)	3153 (82.93%)	
Multipara	17702 (31.35%)	649 (17.07%)	
Family history of DM			0.009
No	54275 (94.53%)	3650 (93.54%)	
Yes	3143 (5.47%)	252 (6.46%)	
GDM			<0.001
No	50016 (87.09%)	3226 (82.68%)	
Yes	7411 (12.91%)	676 (17.32%)	
GDM A2			0.015
No	26956 (96.94%)	1655 (95.89%)	
Yes	852 (3.06%)	7171 (4.11%)	
GDM&PE			<0.001
No	56899 (99.08%)	3831 (98.18%)	
Yes	528 (0.92%)	71 (1.82%)	
LGA			0.869
No	53439 (93.63%)	3617 (93.56%)	
Yes	3638 (6.37%)	249 (6.44%)	

The results indicated that, within the IVF group, the following characteristics were significantly elevated compared to those in the NC group: age, pre-pregnancy BMI, ALT levels, and the proportions of parity, family history of DM, GDM, GDM A2, and gestational diabetes mellitus with preeclampsia. We then assessed these differences after adjusting for potential confounding variables, including age, parity, body mass index, family history of diabetes mellitus, ALT levels, and creatinine levels.

In a multivariable analysis (presented in **Table 2**), we adjusted for confounding factors including age, parity, BMI, family history of DM, ALT levels, and creatinine levels. Our findings indicate that IVF is a significant, albeit modestly associated factor for GDM, with an odds ratio of 1.15 (95% CI: 1.03, 1.28) and a p-value of 0.0148. Following these results, we further investigated potential differences based on factors such as age, BMI, and parity.

**Table 2:** Odds ratio of primary and secondary outcomes.

Exposure	Odds ratio (95% CI)	*p*	Adjust odds ratio (95% CI)	*p*
GDM				
IVF				
No	1		1	
Yes	1.41 (1.30, 1.54)	<0.0001	1.15 (1.03, 1.28)	0.0148
GDM A2				
IVF				
No	1		1	
Yes	1.36 (1.06, 1.74)	0.0154	1.27 (0.96, 1.68)	0.0912
GDM&PE				
IVF				
No	1		1	
Yes	2.00 (1.56, 2.56)	<0.0001	1.30 (0.95, 1.77)	0.0964
LGA				
IVF				
No	1		1	
Yes	1.01 (0.89, 1.15)	0.8691	1.11 (0.96, 1.29)	0.1467

Adjusted for age, parity, body mass index, family history of DM, ALT and creatinine.

The associations between IVF and GDM across different strata of age, BMI, and parity are detailed in **Table 3**. Confounding factors, including age, parity, BMI, family history of diabetes mellitus, ALT levels, and creatinine levels, were adjusted for in the analysis. We observed that IVF was significantly associated with GDM among pregnant women under 35 years of age. This observation aligns with the established cutoff for defining advanced maternal age, which is set at 35 years (16). Our results corroborate with this criterion, further reinforcing the notion that IVF may present specific risks for the development of GDM in younger pregnant women. Similarly, IVF was identified as a contributing factor to GDM among pregnant women with a BMI of less than 24 kg/m². This BMI value delineates the threshold between the upper boundary of normal weight and the onset of overweight (17). Furthermore, in our cohort study, IVF was recognized as a significant factor associated with GDM among women who had never given birth.

**Table 3:** Subgroup analysis of GDM and IVF.

Exposure	Non-adjusted model (95% CI)	*P*	Adjust model (95% CI)	*P*
Age				
<35				
IVF				
No	1		1	
Yes	1.34 (1.19, 1.50)	<0.0001	1.18 (1.02, 1.36)	0.0227
≥35				
IVF				
No	1		1	
Yes	1.18 (1.03, 1.35)	0.0174	1.10 (0.92, 1.31)	0.3190
BMI				
<24				
IVF				
No	1		1	
Yes	1.41 (1.26, 1.57)	<0.0001	1.20 (1.05, 1.37)	0.0063
≥24				
IVF				
No	1		1	
Yes	1.18 (1.00, 1.38)	0.0451	1.03 (0.84, 1.27)	0.7428
Parity				
Primigravida				
IVF				
No	1		1	
Yes	1.51 (1.37, 1.66)	<0.0001	1.16 (1.02, 1.31)	0.0216
Multipara				
IVF				
No	1		1	
Yes	1.19 (0.96, 1.46)	0.1102	1.08 (0.83, 1.41)	0.5769

Adjusted for age, parity, body mass index, family history of DM, ALT and creatinine.

## 4 Discussion

This study conducted a large-sample joint analysis that demonstrated IVF as an independent risk factor for GDM and the risk of GDM among IVF patients increased by 15%. Subgroup analyses further indicated that IVF significantly increased the risk of GDM among women under 35 years of age, those with a pre-pregnancy BMI below 24kg/m², or primigravidae.

### 4.1 Comparison with previous studies

Recent studies have identified IVF as an independent risk factor for GDM. For instance, a prospective cohort study reported a significantly higher incidence of GDM in the IVF group compared to the natural conception group (20% vs. 5.5%) (18). However, another study concluded that ART treatment was not associated with GDM [10], which contrasts with the findings of other research. These discrepancies may be attributed to the relatively small sample sizes and single-center designs of the aforementioned studies, which limit the generalizability and reliability of their results. In contrast, our study, which incorporated a larger sample size and a multicenter collaborative approach, provides more robust and compelling evidence supporting the association between IVF and GDM. Our findings reinforce the conclusion that IVF serves as an independent risk factor for GDM. Large-sample meta-analyses have suggested that the increased risk of GDM associated with IVF may be linked to advanced maternal age (5). However, these studies did not conduct stratified comparisons by age group. In the present study, we utilized 35 years as a threshold to categorize maternal age. Our results indicated that IVF was a significant risk factor for GDM in women under 35 years of age, whereas this association was not significant in women over 35. In the context of IVF, the influence of pre-pregnancy BMI on the risk of GDM remains a topic of ongoing debate. A previous study reported no significant differences in the impact of BMI on obstetric outcomes, including GDM, between women who conceived through IVF and those who conceived naturally (19). In contrast, another study suggested that IVF is an independent risk factor for GDM in overweight women (20). However, our study is the first to demonstrate that IVF significantly increases the risk of GDM among women with a pre-pregnancy BMI below 24. The discrepancies with the first study may be attributed to differences in study populations, particularly the higher incidence of GDM observed in East Asian populations. Compared to the latter study, the current research utilized pre-pregnancy BMI measurements, while the previous study relied on first-trimester BMI data, which may be affected by varying degrees of weight gain during pregnancy. Furthermore, the multi-center design and larger sample size of our study enhance the generalizability and robustness of our findings. Current research on the impact of parity on GDM risk has produced inconsistent findings both domestically and internationally. Some studies have reported no significant association between parity and GDM (21), while others have indicated that multiparous women face a higher risk of GDM compared to primigravidae (22). These discrepancies may be attributed to variations in study populations, including racial diversity, as well as relatively small sample sizes. Notably, we did not identify any studies that clearly demonstrated the influence of parity on GDM risk within the context of IVF. Our study is the first to establish IVF as a significant risk factor for GDM in primiparous women, thereby providing new insights into this understudied area.

### 4.2 Potential mechanisms linking IVF to GDM

The pathophysiological mechanisms linking ART with GDM may be multifactorial, involving procedure-related factors and underlying patient characteristics.

#### 4.2.1 Hormonal alterations during ovarian stimulation

The association between IVF and GDM has been identified, potentially attributable to the supraphysiological levels of estrogen and progesterone during ovarian stimulation, as well as the baseline health status of women. A critical component of the IVF process is controlled ovarian hyperstimulation (COH), which is achieved through the combined administration of multiple hormones to promote follicular development and regulate the endocrine environment. The use of gonadotropins results in the synchronous development of multiple follicles, leading to significant increases in estrogen and progesterone levels in the body. These drastic fluctuations in hormone levels may disrupt insulin signaling pathways, affecting the function of insulin receptors and normal secretion, thereby reducing insulin sensitivity and increasing the risk of diabetes. Elevated progesterone may impair insulin sensitivity via downregulation of GLUT4 expression, while supraphysiological estrogen inhibits insulin receptor substrate phosphorylation—both mechanisms potentially contributing to GDM pathogenesis (23–25). Recent studies have identified an optimal E2/follicle (E2/F) ratio threshold of 246.03 pg/ml on the day of human chorionic gonadotropin (hCG) trigger. For women undergoing IVF treatment, those with an E2/F ratio exceeding this threshold have a significantly lower incidence of GDM (26). Conversely, IVF treatment results in elevated progesterone levels on the hCG trigger day, which can reduce insulin sensitivity by decreasing GLUT4 expression, thereby increasing the risk of GDM compared to women with natural pregnancies (27). Furthermore, metabolic disorders in the body may lead to abnormal regulatory functions of the ovaries, hormonal imbalances, and gonadal dysfunction, which can increase the incidence of infertility (28). Women undergoing IVF treatment often belong to the infertility population and may have underlying metabolic disorders, making them more susceptible to blood glucose regulation imbalances when faced with hormonal changes and metabolic stress during the IVF process.

#### 4.2.2 The role of underlying infertility and shared etiologies

It should be noted that women undergoing IVF often have underlying conditions causing infertility, which may also predispose them to metabolic dysfunction. Polycystic ovary syndrome (PCOS) is the primary cause of anovulatory infertility and has been established as an independent risk factor for insulin resistance and GDM (29). In our retrospective dataset, PCOS diagnosis was not systematically or uniformly documented in obstetric records, thus preventing its inclusion as a covariate in the model. This represents a significant limitation, as residual confounding factors from PCOS and other shared metabolic disorders (e.g., thyroid dysfunction) may influence the observed associations. Although we adjusted for age, BMI, and ALT—proxy variables closely related to metabolic health—the inability to directly account for PCOS necessitates cautious interpretation of the results. Existing literature indicates that even when considering PCOS, IVF remains an independent risk factor for GDM (30). Future research must prospectively collect standardized data on infertility etiology and metabolic comorbidities to distinguish the contributions of underlying diseases from those of the IVF procedure itself.

### 4.3 Interpretation of subgroup findings

#### 4.3.1 Age <35 years and pre-pregnancy BMI <24 kg/m²

IVF was associated with a significantly elevated risk of GDM in women under 35 years of age and those with a pre-pregnancy BMI below 24kg/m². Existing studies have consistently demonstrated strong associations between age, pre-pregnancy BMI, and GDM risk in both naturally conceived and ART-conceived pregnancies (31, 32). Obesity and advancing age are recognized as significant acquired factors contributing to insulin resistance (33, 34).

To reconcile this observation with established pathophysiology, we propose that the risk of developing GDM after IVF is not merely a simple summation of traditional risk factors (such as age and BMI), but rather arises from the interactive effect between metabolic stress induced by the IVF process and an individual’s inherent metabolic stability. Younger women (<35 years) and those with normal pre-pregnancy BMI (<24 kg/m²) generally exhibit higher baseline insulin sensitivity and a more stable metabolic state. In these “lower-risk” subgroups, the exogenous hormonal interventions inherent to IVF may act as a significant metabolic stressor, sufficient to disrupt intrinsic homeostasis.

Conversely, in women at traditionally ‘higher risk’—those aged ≥35 years or with a pre-pregnancy BMI ≥24 kg/m²—the dominant drivers of insulin resistance likely stem from powerful background factors, including age-related declines in insulin clearance, loss of muscle mass, mitochondrial dysfunction, ectopic fat deposition, and chronic low-grade inflammation (35, 36). Consequently, when age is a predominant factor, the impact of IVF on the risk of GDM may be relatively minor and less pronounced.

Regarding BMI, studies suggest that pronounced weight gain has a more significant effect on GDM risk in women with normal weight compared to those who are overweight or obese (37), and pre-pregnancy BMI generally exerts a stronger influence on pregnancy outcomes than gestational weight gain (38). Overweight and obesity are critical acquired factors contributing to insulin resistance, with underlying mechanisms including hypoxia in adipose tissue, inflammatory responses, oxidative stress, elevated free fatty acids, and reduced adiponectin secretion (39, 40). Therefore, while IVF remains a relevant risk factor for GDM, its influence may be less pronounced than that of elevated pre-pregnancy BMI.

#### 4.3.2 Primiparity

IVF was associated with a significantly increased risk of GDM among primiparous women. Primiparous women, who have not previously undergone the metabolic adaptations of pregnancy, may have endocrine and metabolic systems that are more sensitive to perturbation. The hormonal environment associated with IVF may disrupt signaling pathways, including those involved in insulin action, in these individuals. Concurrently, they are initiating adaptation to the insulin resistance induced by placental hormones. The combined effect of IVF-associated hormonal shifts and the physiological insulin resistance of pregnancy may challenge the capacity of pancreatic β-cells in primiparous women to secrete sufficient insulin in a timely manner, leading to difficulties in maintaining glycemic homeostasis (41). Conversely, multiparous women have experienced prior pregnancies, which induce substantial and persistent endocrinological and neurobiological adaptations, such as altered hormonal baselines and enhanced stress resilience, even after parturition (42). These pre-existing adaptations may modulate their physiological response to the stressors of IVF treatment, potentially buffering its impact on metabolic outcomes such as GDM risk.

In conclusion, IVF is associated with an increased risk of GDM, particularly among women under 35 years of age, those with a pre-pregnancy BMI below 24kg/m², or primigravidae, warranting additional clinical attention. IVF may serve as a potential indicator for GDM risk stratification and has significant implications for the management of GDM. This discovery provides valuable evidence to support clinical practice and offers scientifically grounded recommendations for individuals facing infertility. For couples concerned about fertility, we emphasize the importance of seeking timely evaluation and considering earlier attempts at natural conception. This approach may help reduce the likelihood of progressing to infertility treatments such as IVF at an advanced age, which, as our study suggests, may carry an elevated risk of GDM, particularly when IVF is utilized. For women with a BMI below 24kg/m²undergoing IVF treatment, individualized gonadotropin dosing based on ovarian reserve testing may reduce excessive hormonal fluctuations. Additionally, emphasizing weight management in daily life could potentially mitigate the impact of IVF procedures on the incidence of GDM. However, the effectiveness of these proposed measures requires further validation through prospective clinical studies to confirm their feasibility and impact.

### 4.4 Strengths and limitations

This study employed a multicenter, large-sample joint analysis method, which enhances the reliability and representativeness of the results. However, several limitations are present in this study. First, the retrospective design precludes causal inference and is susceptible to residual confounding from unmeasured or imperfectly measured variables, most notably PCOS, specific IVF indications, and detailed lifestyle factors. Second, as previously mentioned, we lacked data on critical IVF treatment parameters (e.g., fresh vs. frozen embryo transfer, stimulation protocols, hormone levels), preventing mechanistic explorations of how different treatment modalities influence GDM risk. Third, information on gestational weight gain and prior GDM history was incomplete, limiting our ability to fully adjust for these relevant factors. Fourth, regarding causal interpretation, our study design precludes dose-response analysis—an important criterion for strengthening causal inference. Both exposure (IVF procedure) and outcome (GDM) are binary variables in our dataset. While the hypothesized mechanism involves ovarian stimulation, we lack continuous, quantifiable metrics of stimulation intensity (e.g., cumulative gonadotropin dose, peak estradiol level per mature follicle) in retrospective clinical records. The absence of such granular treatment data prevents examining whether higher stimulation ‘dose’ correlates with graded increases in GDM risk. This limitation stems from the binary exposure variable and data availability in multicenter obstetric databases, highlighting the need for future prospective studies to capture detailed treatment parameters and better explore potential dose-response relationships.

## 5 Conclusions

In conclusion, our study emphasizes that IVF is a significant risk factor for GDM, particularly among younger women, those with a lower BMI, and primigravidae. This finding underscores the necessity for tailored screening and management strategies for GDM in this population, as they may not be traditionally classified as high-risk according to conventional criteria. These findings highlight the need for a stratified approach to GDM screening in IVF pregnancies and underscore the importance of interdisciplinary collaboration between reproductive and maternal-fetal medicine specialists.

To transition from observed associations to a mechanistic understanding, future prospective studies are warranted. These studies should be specifically designed to collect comprehensive and standardized data on IVF treatment parameters, including ovarian stimulation protocols (e.g., GnRH agonist versus antagonist), gonadotropin dosage, fresh versus frozen embryo transfer cycles, and serial peri-transfer hormone levels (e.g., estradiol, progesterone). Correlating these detailed treatment profiles with dynamic metabolic assessments before, during, and after treatment will be crucial. This integrated approach is essential to disentangle the specific contributions of different IVF protocols to GDM risk, to test the proposed “Metabolic Susceptibility-Stress” hypothesis, and ultimately to develop targeted preventive strategies for at-risk individuals undergoing fertility treatments.

## Data Availability

The original contributions presented in the study are included in the article/supplementary material. Further inquiries can be directed to the corresponding author/s.
